# COL11A1 Polymorphisms Are Associated with Primary Angle-Closure Glaucoma Severity

**DOI:** 10.1155/2019/2604386

**Published:** 2019-01-27

**Authors:** Yani Wan, Shengjie Li, Yanting Gao, Li Tang, Wenjun Cao, Xinghuai Sun

**Affiliations:** ^1^Department of Clinical Laboratory, Eye & ENT Hospital, Shanghai Medical College, Fudan University, Shanghai 200032, China; ^2^Department of Ophthalmology & Visual Science, Eye & ENT Hospital, Shanghai Medical College, Fudan University, Shanghai 200032, China; ^3^State Key Laboratory of Medical Neurobiology, Institutes of Brain Science and Collaborative Innovation Center for Brain Science, Fudan University, Shanghai 200032, China; ^4^NHC Key Laboratory of Myopia, Key Laboratory of Myopia, Chinese Academy of Medical Sciences, and Shanghai Key Laboratory of Visual Impairment and Restoration, Fudan University, Shanghai 200031, China

## Abstract

**Purpose:**

PLEKHA7 and COL11A1 were genotyped for single-nucleotide polymorphisms (SNPs) to investigate the possible association of these two genes with primary angle-closure glaucoma (PACG) and disease severity.

**Method:**

A total of 51 PACG cases and 51 normal controls were recruited. Twelve SNPs in the PLEKHA7 (rs216489, rs1027617, rs366590, rs11024060, rs6486330, rs11024097, and rs11024102) and COL11A1 (rs1676484, rs3753841, rs12138977, rs2126642, and rs2622848) genes were genotyped by direct Sanger sequencing. Distributions of allele frequencies and genotype frequencies in cases and controls, as well as in mild, moderate, and severe subgroups, were compared based on mean defect (MD ≤ 6.00 dB, 6 dB < MD ≤ 12 dB, and MD > 12 dB were considered mild, moderate, and severe, respectively). Independent Student's *t*-tests and chi-square tests were used to compare characteristics of PACG cases and controls. Chi-square tests were used to compare the distribution of allele frequencies in cases and controls and in MD-based subgroups with various degrees of glaucoma severity. Binary logistic regression was used to compare the distribution of genotype frequencies and calculate odds ratios (OR) with confidence intervals (CI).

**Result:**

Three of the 12 SNPs in COL11A1, rs1676486 (*P*=0.026, OR = 2.089, 95% CI = 1.092–3.996), rs3753841 (*P*=0.036, OR = 1.886, 95% CI = 1.038–3.426), and rs12138977 (*P*=0.024, OR = 2.133, 95% CI = 1.104–4.123) were found to have a significant association with PACG. Furthermore, in the subgroup analysis, rs1676486 (*P*=0.018, OR = 2.416, 95% CI = 1.284–4.544; *P*=0.011, OR = 2.119, 95% CI = 1.204–3.729), rs12138977 (*P*=0.009, OR = 2.158, 95% CI = 1.287–3.618; *P*=0.006, OR = 1.962, 95% CI = 1.239–3.106), and rs3753841 (*P*=0.007, OR = 2.550, 95% CI = 1.344–4.839) showed statistically significant differences between moderate/severe groups and controls.

**Conclusion:**

Our data suggested that COL11A1 rs1676484, rs3753841, and rs12138977 polymorphisms may be of value for further study as potential gene-dependent risk factors for developing PACG. Moreover, COL11A1 rs1676484 and rs12138977 polymorphisms might be associated with PACG disease severity.

## 1. Introduction

Primary angle-closure glaucoma (PACG) is a neurodegenerative disease characterized by elevated intraocular pressure (IOP) due to a mechanical obstruction of the trabecular meshwork by either apposition of the peripheral iris to the trabecular meshwork or a synechial closed angle [1]. Epidemiological studies have revealed that East and South-Central Asia had the highest glaucoma burden in 2013. South-Central Asia is expected to experience the steepest increase in the number of glaucoma patients between 2013 and 2040, from 17.06 million to 32.90 million, compared to other Asian subregions [2].

PACG is a complex heterogeneous disease; causative molecular mechanisms leading to PACG are poorly understood, and genetic susceptibility for this disease is still under investigation. Genome-wide association studies (GWAS) have recently identified several new PACG loci and genes, including PLEKHA7, COL11A1, EPDR1, CHAT, GLIS3, FERMT2, and DPM2-FAM102 A, which may shed light on the molecular mechanisms of PACG [3, 4]. Among these genes, COL11A1 and PLEKHA7 may be important. COL11A1 is located on chromosome 1p21, comprises 68 exons, and is approximately 250 kb in length [5]. Pathogenic mutations in this gene can result in type II Stickler syndrome and Marshall syndrome [6, 7], while rs1676486 on COL11A1 is associated with lumbar disc herniation susceptibility [8]. Pleckstrin homology domain-containing family A member 7 (PLEKHA7) is an adherens junction protein [9] required for organizing the epithelial architecture and contributes to tissue homeostasis. Since it is likely involved in regulating fluid flow across the inner wall of the Schlemm's canal [10], it was proposed that mutations in this gene could affect fluid dynamics in the pathophysiology of angle-closure glaucoma [11].

The contribution of the two most associated SNPs (rs11024102 and rs3753841) on PLEKHA7 and COL11A1 was confirmed in patients in Australia, Nepal, and China [11–13]. Moreover, an association was also found between these SNPs and PACG in our previous studies [14]. Using the matrix-assisted laser desorption ionization time-of-flight mass spectrometry method (MALDI-TOF), we genotyped more SNPs in the two genes and found that rs1676486 and rs12138977 in COL11A1, as well as rs216489 and rs11024102 in PLEKHA7, may be associated with an increased risk of PAC/PACG in the Han Chinese population. However, further investigation is needed to determine whether the two genes contribute to different disease stages. In this study, we focused on exploring the association between the PLEKHA7 and COL11A1 SNPs stated above and various degrees of PACG severity.

In this study, we explored the association between 12 SNPs (in PLEKHA7 and COL11A1) and PACG. An analysis was also conducted to determine whether the SNPs are associated with PACG patients experiencing different degrees of disease severity. The association between ocular biometric parameters, such as anterior chamber depth (ACD) and axial length (AL), with 12 SNPs were also investigated. In sum, our work aimed at providing new insights and a better understanding of the association between candidate gene SNPs and disease severity and specific clinical features of PACG.

## 2. Materials and Methods

### 2.1. Patients

The study was approved by the ethical committee of Eye and Ear Nose Throat Hospital at Fudan University in Shanghai, China, and all procedures were conducted in accordance with the Declaration of Helsinki. Written informed consent was obtained from all subjects and normal controls prior to participating in this study.

A total of 51 subjects with PACG and 51 matched healthy controls were consecutively recruited from Eye and Ear Nose Throat Hospital at Fudan University. Each subject underwent a standardized ophthalmic examination performed by glaucoma specialists that included the following assessments: refractive status, slit-lamp biomicroscopy, fundus, IOP, AL, ACD, visual field, and gonioscopy. MD and mean sensitivity (MS) were measured using an automated perimeter (Octopus; Haag-Streit AG, Koeniz, Switzerland). We measured IOP using Goldmann applanation tonometry. Fundus photography was performed with a retinal camera (TRC-NW200, Topcon Corp., Tokyo, Japan). We used an A-scan ultrasound (A-Scan Pachymeter; Ultrasonic, Exton, PA, USA) to measure AL and ACD [15].

### 2.2. Diagnostic and Inclusion Criteria

Our previous study described the PACG inclusion criteria [16], which are detailed below:

1. PACG subjects were selected from hospital inpatients.
2. PACG was diagnosed on the basis of narrow angles with glaucomatous optic neuropathy and corresponding visual field loss. It was defined as a glaucoma hemifield test outside normal limits, including a cluster of three or more noncontiguous points on the pattern deviation plot, not crossing the horizontal meridian with a probability of less than 5% of being present in the age-matched normal (one of which was less than 1%), an abnormal pattern standard deviation with a *P* value less than 5% occurring in the normal population, and fulfilling the test reliability criteria (i.e., fixation losses less than 20%, false positives less than 33%, and false negatives less than 33%). PACG was diagnosed in eyes meeting the following criteria: narrow angles and elevated IOP (IOP > 21 mm·Hg); at least 180 degrees of angle-closure obliterating the pigmented part of the trabecular meshwork, whether synechial or appositional, segmented or continuous; and eyes in which the degree of the peripheral anterior synechiae is too extensive to be managed by laser peripheral iridotomy [17–19]. Subjects receiving glaucoma medications were also included.
3. PACG and control subjects had no major systemic diseases (e.g., autoimmune disease or cancer) that could likely confound the results of the study. Participants with secondary angle-closure glaucoma due to uveitis, trauma, neovascularization, or any other optic nerve injury affecting either eye were excluded.

### 2.3. Blood Sample Collection and Detection

Whole blood samples (2 ml each) were collected from each subject, which was used for DNA isolation and an analysis of the selected genetic polymorphisms. Before treatment, all the samples were stored in an EDTA tube (Wuhan Zhiyuan Medical Technology Co., Ltd., Wuhan, China) and stored in a −80°C refrigerator.

### 2.4. Experimental Procedure

Genomic DNA was extracted from leukocytes of peripheral blood drawn from each participant. A RelaxGene Blood DNA System (0.1–20 ml) (DP349) was used to purify the blood. Twelve SNPs (i.e., rs1676486, rs3753841, rs12138977, rs2126642, and rs2622848 in COL11A1 and rs216489, rs1027617, rs366590, rs11024060, rs6486330, rs11024097, and rs11024102 in PLEKHA7), including two SNPs previously reported as significantly associated with PACG through GWAS (i.e., rs3753841 and rs11024102), were genotyped. The SNPs chosen in this study were either tagging or coding SNPs with minor allele frequencies greater than 10% in Chinese Han populations referenced to the HapMap database (http://hapmap.ncbi.nlm.nih.gov/, in the public domain).

Briefly, the polymerase chain reactions (PCRs) contained 50 ng of genomic DNA, 1 *μ*L of each primer (10 *μ*mol/L), 1 *μ*L of 10x buffer (Qiagen, Hilden, Germany), 0.2 *μ*L of MgCl_2_ (25 mM; Qiagen, Inc.), 0.2 *μ*L of deoxyribonucleotide triphosphates (10 mM; Shanghai Yeasen Biological Technology Co., Ltd., Shanghai, China), and 0.06 *μ*L of HotStarTaq DNA polymerase (5 U/*μ*L; Qiagen, Inc.). Amplified PCR products were separated by agarose gel electrophoresis and visualized by staining with YeaRed nucleic acid gel stain (10000x in water, Shanghai Yeasen Biological Technology Co., Ltd., Shanghai, China). PCRs were performed on an S1000™ Thermal Cycler (Bio-Rad Laboratories, Inc., California, US). The PCR cycling program was 15 min at 95°C, 11 cycles × (94°C 20 s, 62°C–0.5°C/cycle 40 s, 72°C 1 min), 24 cycles × (94°C 20 s, 57°C 30 s, 72°C 1 min), 2 min at 72°C, and 4°C permanently. All the SNPs were genotyped via direct Sanger sequencing.

### 2.5. Data Analysis

The data were analyzed using standard statistical software (IBM SPSS, version 24.0; IBM, Inc., New York, USA). Results are presented as mean ± SD. Independent Student's *t*-tests and chi-square tests were used to compare patient characteristics between groups. The chi-square test was used to evaluate the Hardy–Weinberg equilibrium (HWE) and differences in allele frequencies for each SNP between the case and control groups. Logistic regression was used to compare the distribution of genotype frequencies and calculate odds ratios (OR) with 95% confidence intervals (CI) and adjust for age and sex. A chi-square test was also used to analyze subgroups in which patients were divided into three groups with different degrees of severity based on perimetry: mild (MD ≤ 6.00 dB), moderate (6 dB < MD ≤ 12 dB), and severe (MD > 12 dB) [20, 21]. A linear regression model was used for association testing between genotypes and ACD or AL as quantitative traits. A value of *P* < 0.05 was considered statistically significant.

## 3. Results

### 3.1. Characteristics of the Study Participants

A total of 51 subjects with PACG (32 females, 19 males) and 51 normal controls (34 females, 17 males) were enrolled in this study. The mean age of cases was 59.29 ± 11.9 years and 58.24 ± 6.49 years for controls. One eye was randomly selected if both eyes suffered from PACG. There were a total of 51 eyes in the PACG group. There was no statistical difference in the mean age, SBP, DBP, or sex distribution between PACG and control subjects (*P* > 0.05). Demographics and clinical characteristics of cases and controls are shown in Table 1. All twelve SNPs were successfully genotyped, and their allele distributions were within the Hardy–Weinberg equilibrium (HWE) for both the case and control group (*P* > 0.05; Table 2).

### 3.2. SNP Allele/Genotype Frequencies and Associations for 12 SNPs between Cases and Controls

Among the 12 SNPs, a significant genetic association was identified for rs1676486 (*P*=0.026, OR = 2.089, 95% CI = 1.092–3.996), rs3753841 (*P*=0.036, OR = 1.886, 95% CI = 1.038–3.426) and rs12138977 (*P*=0.024, OR = 2.133, 95% CI = 1.104–4.123) in COL11A1, with higher minor allele frequencies found in cases than in controls (Table 3). As shown in Table 4, AG + GG genotype frequencies of rs1676486 were significantly higher in cases than in controls (*P*=0.031, OR = 2.405, 95% CI = 1.086–5.332). A significant association was also identified for GG genotype frequencies of rs3753841 (*P*=0.040, OR = 5.684, 95% CI = 1.084–29.803). CT + CC genotype frequencies of rs12138977 (*P*=0.03, OR = 2.417, 95% CI = 1.088–5.368) also showed statistically significant evidence of genetic association in analyses comparing PACG and controls.

### 3.3. SNP Allele Frequencies and Associations for the Top 3 SNPs in Subgroup Analyses versus Controls

The 51 patient cases were categorized into 3 different severity subgroups according to MD: 22 were classified as mild, 10 as moderate, and 19 as severe. There were no statistical differences in the mean age or gender among the three groups. In the subgroup analysis of moderate PACG versus controls, three SNPs, including rs1676486 (*P*=0.018, OR = 2.416, 95% CI = 1.284–4.544), rs3753841 (*P*=0.009, OR = 2.158, 95% CI = 1.287–3.618), and rs12138977 (*P*=0.007, OR = 2.550, 95% CI = 1.344–4.839), showed a statistically significant association. Additionally, the following SNPs showed a statistically significant association between severe PACG and controls: rs1676486 (*P*=0.011, OR = 2.119, 95% CI = 1.204–3.729), rs3753841 (*P*=0.006, OR = 1.962, 95% CI = 1.239–3.106), and rs1213897 (*P*=0.001, OR = 2.535, 95% CI = 1.465–4.386). However, when the mild PACG subgroup was compared to controls, there was no statistically significant association (Table 5).

### 3.4. Association Analysis Results of 12 SNPs with AL and ACD

In the linear regression model testing of PACG genotypes with AL and ACD, no significant association was observed between SNPs and either AL or ACD. Association results for the 12 SNPs are illustrated in Table 6.

## 4. Discussion

We conducted a candidate gene association study of 12 SNPs on two PACG susceptibility genes and anatomical quantitative trait risk factors for angle closure (ACD and AL) using Sanger sequencing. In our study, rs1676486, rs3753841, and rs12138977 in COL11A1 showed significant differences in minor allele and genotype frequencies between cases and controls (see Tables 3 and 4), which suggests that carriers of the minor alleles of these SNPs are approximately twice as likely to develop PACG (see Table 3). Additionally, none of the examined SNPs showed a significant association with either ACD or AL, which suggests the disease mechanisms act independently of a shallower anterior chamber and shorter eyeball length. A notable strength of this study is that it demonstrated the association between PACG loci and disease severity.

Previous studies identified rs3753841 in COL11A1 and rs11024102 in PLEKHA7 as genetic risk factors for PACG [3, 4]. The involvement of genetic risk factors in disease pathogenesis can be better understood by studying their association in an independent cohort. Despite the genotyping technology difference, the results of our study are comparable to previously reported values and our team's previous conclusions [3, 14]. In a previous GWAS [3], rs3753841, a coding SNP with an amino acid change of proline to leucine, was the highest associated SNP in the COL11A1 gene. In our study, however, rs12138977 showed the most significant association with PACG (*P*=0.024).

Moreover, this study appeared to be the first to examine the association of susceptibility loci with MD-based glaucoma severity subgroups. It is well known that clarifying risk factors for different stages of a disease can be helpful for early diagnosis and prognosis in clinical practice. Surprisingly, we found that the allele frequency distribution of the three SNPs discussed above (i.e., rs1676484, rs3753841, and rs12138977) was significantly different in moderate/severe PACG and controls, which suggested they may be associated with PACG disease severity. Thus, these three SNPs might be clinically useful in predicting PACG progression.

COL11A1 encodes the alpha-1 chain of type Xl collagen, a member of the collagen family and a major component of the interstitial extracellular matrix (ECM) [22]. ECM material in aqueous humor outflow pathways is thought to be essential for the generation of outflow resistance that induces IOP [22]. Both rs1676486 and rs3753841 are located in the coding region of COL11A1, and each of them consists of two different amino acid coding patterns. It was also reported that the COL11A1 gene was upregulated in the lamina cribrosa of glaucoma patients, which further indicates the involvement of the expression and regulation of ECM genes in glaucoma pathogenesis [23]. We supposed that various amino acid residuals of these two sites and their combination probably affect the configuration and function of collagens and even the ECM, and the conventional outflow pathway, may be changed as a result. However, COL11A1 is associated with type II Stickler and Marshall syndromes, which are congenital conditions that include high myopia and blindness due to retinal detachment [6, 7, 24]. In a recent case report [25], a child with Stickler syndrome type II was found to have a novel missense mutation in COL11A1. The child was diagnosed with retinal dysplasia and persistent hyperplastic primary vitreous (PHPV) of the right eye after birth and later developed closed-angle glaucoma in the same eye that did not respond to medical therapy. The above studies further illustrated that COL11A1 in ECM may be associated with pathological processes involved in angle closure. Additional studies are required to understand the exact role of polymorphisms in the pathogenesis of glaucoma.

Our study suffered several limitations. Due to the strict inclusion criteria, this study's sample size was relatively small. Additionally, although associations between PACG severity and COL11A1 polymorphisms were observed, the exact mechanism remains unknown and needs to be further investigated.

Overall, our study validates the findings of GWAS implicating COL11A1 in the pathogenesis of PACG. The results revealed significant associations between rs1676486, rs3753841, and rs12138977 polymorphisms and PACG. Moreover, carriers of the minor alleles of these SNPs were shown to confer significant risk for PACG. We also demonstrated these three SNPs may be associated with disease severity. Further studies will be necessary to investigate how the candidate genes affect the pathogenicity of angle-closure glaucoma.

## Figures and Tables

**Table 1:** Demographics and clinical characteristics of subjects.

Characteristics	Cases	Controls	*T* value/chi-square	*P* value
Number	51	51		
Age	59.29 ± 11.9	58.24 ± 6.49	0.558	0.579
Female/male	32/19	34/17	0.172	0.679
VCDR	0.61 ± 0.23			
IOP (mmHg)	22.45 ± 9.77			
ACD	1.98 ± 0.45			
AL	21.68 ± 3.08			
MD	12.86 ± 9.63			
MS	14.91 ± 9.59			
SBP	133.00 ± 13.36	129.31 ± 17.53	1.182	0.240
DBP	78.31 ± 7.42	79.90 ± 7.81	−1.039	0.300
HTN (yes/no)	19/32	14/37	1.120	0.290
DM (yes/no)	49/2	48/3	0.210	0.647

Data are presented as mean ± SD. The independent Student's *t*-test and chi-square test were used. VCDR: vertical cup-disc ratio; IOP: intraocular pressure; ACD: anterior chamber depth; AL: axial length; MD: mean defect; MS: mean sensitivity; SBP: systolic blood pressure; DBP: diastolic blood pressure; HTN: hypertension; DM: diabetes mellitus.

**Table 2:** Hardy–Weinberg equilibrium.

Gene	SNP	Cases (*N* = 51)		Controls (*N* = 51)	
		Chi-square	*P*	Chi-square	*P*
COL11A1	rs1676486	1.11	0.57	2.16	0.33
	rs3753841	0.06	0.97	0.5	0.80
	rs12138977	0.94	0.66	1.96	0.40
	rs2126642	0.05	0.98	1.02	1.00
	rs2622848	0.65	0.72	0.28	0.87

PLEKHA7	rs216489	0.14	0.93	0.84	0.66
	rs1027617	0.10	0.95	0.6	0.74
	rs366590	0.11	0.95	3.28	0.19
	rs11024060	0.08	0.96	1.92	0.38
	rs6486330	3.28	0.19	0.05	0.98
	rs11024097	3.97	0.14	0.6	0.74
	rs11024102	0.37	0.83	<0.01	1.00

**Table 3:** Single-nucleotide polymorphism allele frequencies and associations for 12 SNPs between cases and controls.

SNP	CHR	BP	MA	MAF case	MAF control	*P* values	OR (95% CI)
rs1676486	1	103354138	A	0.32	0.19	0.026	2.089 (1.092–3.996)
rs3753841	1	103379918	G	0.39	0.26	0.036	1.886 (1.038–3.426)
rs12138977	1	103393457	C	0.31	0.18	0.024	2.133 (1.104–4.123)
rs2126642	1	103405793	A	0.11	0.10	0.818	1.112 (0.450–2.746)
rs2622848	1	103421003	C	0.10	0.07	0.449	1.475 (0.539–4.040)
rs216489	11	16823736	G	0.41	0.38	0.668	1.131 (0.645–1.982)
rs1027617	11	16842787	A	0.38	0.45	0.321	0.754 (0.431–1.317)
rs366590	11	16872440	A	0.23	0.20	0.607	1.194 (0.608–2.343)
rs11024060	11	16881961	T	0.29	0.36	0.297	0.732 (0.407–1.316)
rs6486330	11	16990290	T	0.20	0.19	0.859	1.065 (0.530–2.142)
rs11024097	11	16999029	C	0.36	0.45	0.200	0.693 (0.395–1.215)
rs11024102	11	17008605	C	0.44	0.41	0.671	1.128 (0.647–1.965)

Chi-square tests and logistic regression were used. CHR: chromosome; BP: base pair position; MA: minor allele; MAF: minor allele frequency; MAF Case: minor allele frequencies in cases; MAF Control: minor allele frequencies in controls; OR: odds ratio; CI: confidence interval.

**Table 4:** Single nucleotide polymorphism genotype frequencies and associations for 12 SNPs between cases and controls.

SNP	Genotype	Case		Control		*P* values	OR (95% CI)
		*N*	%	*N*	%		
rs1676486	GG	21	41.20	32	62.70	1	
	AG	27	52.90	19	37.3	0.060	2.165 (0.968–4.842)
	AA	3	5.88	0	0	—	—
	AG + AA	30	58.8	19	37.3	0.031	2.406 (1.086–5.332)

rs3753841	AA	19	37.3	27	52.9	1	
	AG	24	47.1	22	43.1	0.297	1.550 (0.680–3.534)
	GG	8	15.7	2	3.92	0.040	5.684 (1.084–29.803)
	AG + GG	32	72.4	24	47.1	0.11	0.528 (0.239–1.163)

rs12138977	TT	22	43.1	33	64.7	1	
	CT	26	51.0	18	35.3	0.061	2.167 (0.966–4.859)
	CC	3	5.9	0	0	—	—
	CT + CC	29	56.9	18	35.3	0.030	2.417 (1.088–5.368)

rs2126642	GG	40	78.4	42	82.4	1	
	AG	11	21.6	8	15.7	0.475	1.444 (0.527–3.958)
	AA	0	0	1	2.0	—	—
	AG + AA	11	21.6	9	17.6	0.618	1.283 (0.481–3.425)

rs2622848	TT	42	82.4	44	86.3	1	
	CT	8	15.7	7	13.7	0.748	1.197 (0.399–3.593)
	CC	1	2.0	0	0	—	—
	CT + CC	9	17.6	7	13.70	0.587	1.347 (0.460–3.944)

rs216489	AA	17	0.33	21	0.41	1	
	AG	26	0.51	21	0.41	0.333	1.529 (0.647–3.614)
	GG	8	0.16	9	0.18	0.873	1.098 (0.349–3.458)
	AG + GG	34	0.67	30	0.59	0.413	1.4 (0.625–3.135)

rs1027617	GG	20	0.39	14	0.27	1	
	AG	23	0.45	28	0.55	0.427	1.607 (0.498–5.188)
	AA	8	0.16	9	0.18	0.888	0.924 (0.307–2.778)
	AG + AA	31	0.61	37	0.73	0.209	0.586 (0.255–1.349)

rs366590	GG	31	0.61	35	0.69	1	
	AG	17	0.33	12	0.24	0.297	1.599 (0.661–3.868)
	AA	3	0.06	4	0.08	0.836	0.847 (0.176–4.083)
	AG + AA	20	0.39	16	0.31	0.408	1.411 (0.624–3.192)

rs11024060	CC	25	0.49	23	0.45	1	
	CT	22	0.43	19	0.37	0.882	1.065 (0.462–2.456)
	TT	4	0.08	9	0.18	0.18	0.409 (0.111–1.511)
	CT + TT	26	0.51	28	0.55	0.692	1.171 (0.538–2.549)

rs6486330	CC	35	0.69	34	0.67	1	
	CT	12	0.24	15	0.29	0.58	0.777 (0.318–1.9)
	TT	4	0.08	2	0.04	0.46	1.943 (0.334–11.313)
	CT + TT	16	0.31	17	0.33	0.832	0.914 (0.399–2.097)

rs11024097	TT	24	0.47	14	0.27	1	
	CT	17	0.33	28	0.55	0.039	0.391 (0.161–0.953)
	CC	10	0.2	9	0.18	0.493	0.676 (0.221–2.071)
	CT + CC	27	0.53	37	0.73	0.066	0.461 (0.202–1.052)

rs11024102	TT	17	0.33	18	0.35	1	
	CT	23	0.45	24	0.47	0.898	1.059 (0.443–2.531)
	CC	11	0.22	9	0.18	0.776	1.176 (0.385–3.599)
	CT + CC	34	0.67	34	0.67	0.835	1.091 (0.482–2.471)

Logistic regression was used.

**Table 5:** SNP allele frequencies and associations for the top 3 SNPs in subgroup analysis of mild/moderate/severe PACG cases versus controls.

SNP	CHR	MA	MAF control	Mild (*N* = 22)			Moderate (*N* = 10)			Severe (*N* = 19)		
				MAF	*P*	OR (95% CI)	MAF	*P*	OR (95% CI)	MAF	*P*	OR (95% CI)
rs1676486	1	A	0.19	0.2	0.797	1.098 (0.540–2.233)	0.45	0.018	2.416 (1.284–4.544)	0.39	0.011	2.119 (1.204–3.729)
rs3753841	1	G	0.26	0.23	0.722	0.892 (0.471–1.687)	0.55	0.009	2.158 (1.287–3.618)	0.5	0.006	1.962 (1.239–3.106)
rs12138977	1	C	0.18	0.14	0.549	0.773 (0.329–1.814)	0.45	0.007	2.550 (1.344–4.839)	0.45	0.001	2.535 (1.465–4.386)

Chi-square tests and logistic regression were used.

**Table 6:** Association results of 12 SNPs with axial length and anterior chamber depth.

SNP	CHR	MA	ACD		AL	
			Beta	*P*	Beta	*P*
rs1676486	1	A	0.145	0.372	0.161	0.328
rs3753841	1	G	0.223	0.167	0.28	0.084
rs12138977	1	C	0.211	0.192	0.199	0.223
rs2126642	1	A	0.26	0.105	0.174	0.289
rs2622848	1	C	0.132	0.415	0.14	0.39
rs216489	11	G	−0.181	0.264	−0.053	0.749
rs1027617	11	A	−0.169	0.298	0.164	0.319
rs366590	11	A	0.809	0.04	0.117	0.477
rs11024060	11	T	−0.112	0.492	0.186	0.257
rs6486330	11	T	0.003	0.984	0.08	0.629
rs11024097	11	C	−0.101	0.533	−0.049	0.766
rs11024102	11	C	−0.008	0.96	−0.003	0.983

Liner regression was used. ACD: anterior chamber depth; AL: axial length.

## Data Availability

The data used to support the findings of this study are available from the corresponding author upon request.
